# Longitudinal Analysis of Fluoride Levels in Irish Water Supplies: A 52‐Year Review

**DOI:** 10.1111/cdoe.70055

**Published:** 2026-01-28

**Authors:** Vinay Sharma, Brian O'Connell, Eithne O'Flaherty, Jan Rigby, Jacinta McLoughlin, Michael Cronin, Michael O'Sullivan, Lewis Winning, Oscar Cassetti, Michael Crowe

**Affiliations:** ^1^ Division of Restorative Dentistry and Periodontology Dublin Dental University Hospital, Trinity College Dublin Dublin Ireland; ^2^ National Centre for Geocomputation (NCG) Maynooth University Maynooth Ireland; ^3^ School of Mathematical Sciences University College Cork Cork Ireland

**Keywords:** community water fluoridation, fluoride, Ireland, public water supplies

## Abstract

**Background/Aim:**

The Health (Fluoridation of Water Supplies) Act of 1960 in Ireland mandates monthly fluoride sampling in Public Water Supplies (PWS). In 2007, authorities adjusted the mandated fluoride concentration from 0.8–1.0 to 0.6–0.8 mg/L. Approximately 71% of the Irish population has access to fluoridated drinking water. This study aimed to analyse fluoride measurements in Irish water supplies for five decades (1964–2016) to assess compliance and effectiveness of the fluoridation programme.

**Methods:**

Data were sourced from government records and Environmental Protection Agency (EPA) reports. Analysis focused on fluoride concentration measurements, compliance rates, and data completeness across public, private, and group water supplies. Descriptive statistics were used to evaluate trends and patterns in fluoride levels over time.

**Results:**

By 2000, over 90% (*n* = 307) of PWS, each serving more than 1000 persons, were fluoridated. In the early monitoring period (1964–69), missing data were substantial at 66%, with *satisfactory* fluoride results (0.8–1.00 mg/L) at only 17% and *marginal* results (0.70–0.80 and > 1.00–1.10 mg/L) at 15%. Compliance improved steadily, reaching peak performance in 1994–99 with 57% *satisfactory* results. Following the 2007 adjustment in target concentrations, missing data decreased significantly to 18%, with *satisfactory* results (0.60–0.80 mg/L) increasing from 40% to 49% and *marginal* results (0.50–0.60 mg/L and > 0.80–0.90 mg/L) stabilising at 7%–13%. Analysis of private and group supplies revealed evolving trends: from 2000 to 2006, 21% of fluoride testing results were *satisfactory* and 75% *marginal*, while the 2007–2016 period showed 39% *satisfactory* and 48% *unsatisfactory* results, though only 1% exceeded 0.9 mg/L.

**Conclusion:**

The fluoride control in PWS has been largely effective, with consistent improvements in monitoring practices and compliance with target levels over the study period.

**Abbreviations**
CWF: community water fluoridation
EPA: Environmental Protection Agency
EU: European Union
GWS: group water supplies
HSE: Health Service Executive
PRG: group water supplies with a private source
PRI: private water supplies
PUG: group supplies with public sources
PWS: public water supplies
USPHS: United States Public Health Service
WHO: World Health Organisation

## 1 Introduction

Community water fluoridation (CWF) involves the addition of fluoride compounds to Public Water Supplies (PWS) to achieve a given target concentration to improve dental health [1]. Recognised as one of the Ten Great Public Health Achievements of the 20th Century [2], CWF has been implemented globally across 25 countries, serving an estimated 400 million people [3]. The United States maintains the most extensive fluoridation program, with approximately 63% (209 million individuals) receiving fluoridated water [4]. Approximately 3% of the Western European population, in Ireland and selected regions of the UK and Spain, receives fluoridated drinking water [1, 3].

Ireland is the only European country with mandatory legislation requiring the fluoridation of PWS [5]. This legislative framework also mandates daily fluoride monitoring at treatment plants (operational monitoring) and monthly testing of fluoride samples collected from representative consumer sites by Environmental Health Officers from the Health Service Executive (HSE) and sent to Public Analysts Laboratories (compliance monitoring) in accordance with the European Communities (Drinking Water) Regulations 2000 [5, 6]. Currently, approximately 71% of the population in Ireland receives fluoridated drinking water [7].

International drinking water fluoride limits (maximum allowable concentration that must not be exceeded) and standards (enforceable regulatory requirements adopted by competent authorities) vary considerably across jurisdictions. The World Health Organisation (WHO) recommends a maximum of 1.5 mg/L fluoride in drinking water, emphasising that “the volume of water consumed and intake from other sources should be considered when setting national standards” [8]. Under the European Communities (Drinking Water) Regulations 2000, which implemented EU Directive (No. 98/83/EC) during the present study period, the European Union permitted up to 1.0 mg/L of fluoride for fluoridated supplies and 1.5 mg/L for naturally occurring fluoride [9]. The most recent EU (Drinking Water) regulations 2023, which implement the EU drinking water directive, apply a single maximum allowable concentration (parametric value) of 1.5 mg/L of fluoride in drinking water intended for human consumption, with no distinction between fluoridated and non‐fluoridated supplies [10]. In Ireland, the Environmental Protection Agency (EPA) enforces a national fluoride standard of 0.8 mg/L for fluoridated water supplies and 1.5 mg/L for supplies with naturally occurring fluoride [11]. This contrasts with the United States, where the EPA's drinking water standard for fluoride is 4.0 mg/L (primary maximum contaminant level (MCL)), with a secondary guideline of 2.0 mg/L [12].

In Ireland, separate from drinking water quality standards, the Fluoridation of Water Supplies Regulations 2007 establish operational requirements for deliberately fluoridated public water supplies [5]. The current target concentration (an optimal concentration set to achieve a public‐health benefit) for CWF is 0.7 mg/L, with an accepted performance range of 0.6–0.8 mg/L for both operational and compliance monitoring samples. The target concentration and accepted performance range were lowered in 2007 from 1.0 mg/L (range 0.8–1.0 mg/L) to reduce dental fluorosis risk while maintaining caries prevention efficacy [7, 11]. The Irish 2007 policy adjustment aligned with international trends toward lower optimal levels. The United States Public Health Service (USPHS) similarly reduced its recommended target concentration of fluoride to a uniform 0.7 mg/L in 2015, reflecting evidence of increased fluoride availability from multiple sources, including toothpastes, supplements, and mouth rinses, combined with a lack of correlation between climate and water consumption [13, 14]. Other countries implementing 0.7 mg/L CWF targets include Canada [15], New Zealand [16] and Brazil [17]. While Singapore has further reduced the recommended target fluoride levels from 0.7 to 0.5 mg/L [18], in Australia, a target range of 0.5–1.0 mg/L is recommended for CWF programmes, depending on the climate [14].

Since the introduction of CWF in the 1960s, fluoride dosing pumps at water treatment plants have been used to add fluoride to treated water. Initially, the process was supervised by humans and mechanically controlled. However, over time, operations shifted toward more automated systems (e.g., flow‐proportional dosing) [19, 20]. Three chemicals used in water fluoridation are sodium fluoride, sodium silicofluoride, and hydrofluosilicic acid. In the early years of fluoridation, all three chemicals were used in Ireland. However, after a few years, all water treatment plants began using hydrofluosilicic acid at a strength of 10.9% [5, 19].

Drinking water in Ireland originates from groundwater, surface water (including rivers and lakes), and springs. Public water supplies mostly use surface water sources, while private group and private supplies depend on groundwater and spring water [21]. Water supply system in Ireland comprises multiple sources with varying fluoridation requirements and oversight (Table S1, Data S1). Uisce Éireann (formerly Irish Water) manages 962 PWS serving 83.3% of the Irish population [11]. Group and private supplies, serving the remaining population, are exempt from fluoridation legislation but remain subject to quality monitoring requirements under drinking water regulations [22]. This bifurcated system creates potential disparities in fluoride exposure across different population segments. Uisce Éireann (Irish Water) is responsible for monitoring of fluoride levels in Irish PWS in accordance with Fluoridation of Water Supplies Regulations 2007 (S.I. No. 42 of 2007) and European Communities (Drinking Water) Regulations, 2023 (S.I. No. 99 of 2023) [10, 23]. However, local authorities often monitor group and private water supplies on behalf of their owners and trustees [21]. Previous analyses of the water fluoride levels in Irish PWS have examined monthly distillation records [22, 24, 25]. There have been gradual improvements in both result availability and compliance with national recommended limits since water fluoridation was introduced. From 2013 to 2015, the proportion of samples exceeding the national standard of 0.8 mg/L, as reported by the EPA, ranged between 0.9% and 1.3% [11, 21].

To our knowledge, detailed fluoride monitoring data are not publicly available. This study addresses this gap by analysing water fluoride testing results across all water supply types in Ireland over five decades (1964–2016), providing the first comprehensive evaluation of compliance patterns, temporal trends, and disparities in fluoride access. Specific objectives include: (1) characterising long‐term compliance with statutory fluoride limits; (2) identifying temporal and geographic variations in water fluoride concentrations in fluoridated PWS; (3) assessing fluoride access disparities between public, group and private water supplies; and (4) informing policy recommendations for optimising fluoridation programme effectiveness and equity.

## 2 Methodology

### 2.1 Data Source

Fluoride monitoring data were systematically collected from multiple authoritative sources spanning 1964–2016. Primary data sources included: (1) archived monthly distillation test reports maintained by the Department of Health; (2) historical surveillance reports from the Society of Chief and Principal Dental Surgeons; (3) Environmental Health Officer compliance records from local health authorities; and (4) published Environmental Protection Agency reports (2000–2016). A standardised data extraction form was developed, and the following information was extracted: water supply name, county, start date of fluoridation, end date of fluoridation, water supply code, fluoride results source, population size served and monthly fluoride testing results (in mg/L). The water supply identification and fluoridation status were validated using multiple sources, including Statutory Instruments recording fluoridation commencement dates, consultation with retired Environmental Health Officers and Principal Dental Surgeons with institutional knowledge of historical monitoring practices, and verification against water utility records, where available.

### 2.2 Study Outcome and Classification

Monthly fluoride concentration in Public Water Supplies (PWS) was the primary analysis metric. To ensure consistency with the most comprehensive Irish fluoridation evaluation available and enable comparison with policy benchmarks, this study modified the three‐tier classification system from the 2002 Department of Health and Children fluoridation evaluation [22] and classified results as *satisfactory*, *marginal*, *unsatisfactory low*, and *unsatisfactory high*. The classification thresholds were adjusted to reflect the appropriate statutory limits for each era (Table 1).

**TABLE 1:** Classification of monthly fluoride test results, adapted from McLoughlin, Clarkson [22].

Classification of monthly fluoride test results	1960s–2006 (Statutory limit 0.8–1.0 mg/L fluoride)	2007–2016 (Statutory limit 0.6–0.8 mg/L fluoride)
Satisfactory	(0.80–1.00)	(0.60–0.80)
Marginal	(0.70 < 0.80) & (> 1.00 ≤ 1.10)	(0.50 < 0.60) & (> 0.80 ≤ 0.90)
Unsatisfactory low	(< 0.70)	(< 0.50)
Unsatisfactory high	(≥ 1.11)	(≥ 0.90)

*Note:* Statutory compliance standards apply only to fluoridated public water supplies and are not applicable to non‐fluoridated public, group or private supplies. For the purpose of comparative classification of fluoride concentrations, these thresholds were applied consistently across all water supply types, including public, group and private water schemes.

This classification system was selected as it represents the most comprehensive categorisation framework available for Irish fluoride monitoring data, incorporating both under‐ and over‐fluoridation risks through separate unsatisfactory categories. This classification approach differs from earlier publications that used simpler categories [24, 25] and EPA reports that focused only on exceedances [11]. For comparative classification of fluoride concentrations across all water supply types, these thresholds were applied uniformly; however, the compliance parameters themselves are legally applicable only to fluoridated public water supplies and do not constitute regulatory standards for non‐fluoridated public, group, or private schemes. PWS were categorised by population served using six size categories, based on the European Communities Drinking Water Regulations (S.I. 278 of 2007) and EPA guidelines [6], consistent with international research [26]: < 50, 50–499, 500–4999, 5000–9999, 10 000–19 999, and ≥ 20 000 people.

### 2.3 Data Management and Analysis

Data from historical records and EPA reports were compiled and validated in Excel spreadsheets, with inconsistencies resolved through consultation with subject matter experts. To ensure clarity and avoid duplication, duplicate public water supply names were assigned unique identifiers. Historical documents documented fluoridation commencement dates for PWS; where commencement dates were not specified, the date of the first available result was designated as the commencement date. Missing data patterns were documented but not imputed, as the reasons for historical absences (staff shortages, untrained personnel, administrative gaps) were not documented and precluded reliable statistical modelling. All analyses were conducted using R statistical software [27]. For continuous variables, measures of central tendency (mean and median) and dispersion (standard deviation [SD] and interquartile range [IQR]) were calculated. For categorical variables, absolute counts and relative proportions (%) were presented. Temporal trends were assessed using descriptive time‐series analysis (chi‐square test) and visual inspection. The impact of the 2007 regulatory change was evaluated by comparing fluoride concentrations and compliance rates between the pre‐2007 (1960s–2006) and post‐2007 (2007–2016) periods. Data distributions were visualised using time‐series plots for continuous variables and stacked bar charts for categorical compliance data. Statistical significance was established at *p* < 0.05. Given the substantial missing data (12%) and unknown missingness mechanisms in historical records, results should be interpreted as descriptive trends rather than definitive population estimates.

### 2.4 Ethics and Patient and Public Involvement

Ethical approval was not required for this secondary analysis of water fluoride monitoring data collected through routine public health surveillance. No personal or identifiable information was involved in this study.

## 3 Results

### 3.1 Data Availability

Complete longitudinal data (1960s–2016) were available for 336 PWS, predominantly serving populations of more than 1000 people. By 2000, over 90% (*n* = 307) of PWS were fluoridated. An additional 634 PWS had insufficient data for longitudinal analysis (average 8 monthly results over 16 years, 2000–2016) and were excluded from longitudinal analysis (Figure S1, Data S1). Fluoride testing results for group water supplies (public and private sources) and private supplies were available from EPA reports for 2000–2016 only (Table 2).

**TABLE 2:** Water supply type, count and temporal coverage of available data for analysis.

Supply type	Total monthly fluoride results	Number of unique supplies	Temporal coverage of available data
Public water supply (PWS)	129 831	336	1964–2016
Public water supply (PWS)	5408	634	2000–2016
Group water supply (GWS, could be private or public)	338	275	2000–2016
Group water supply with a private source (PRG)	3058	681	2000–2016
Private water supply (PRI)	423	169	2000–2016
Group water supply with a public source (PUG)	3195	684	2000–2016

### 3.2 Public Water Supplies

#### 3.2.1 Data Quality

**FIGURE 1:**
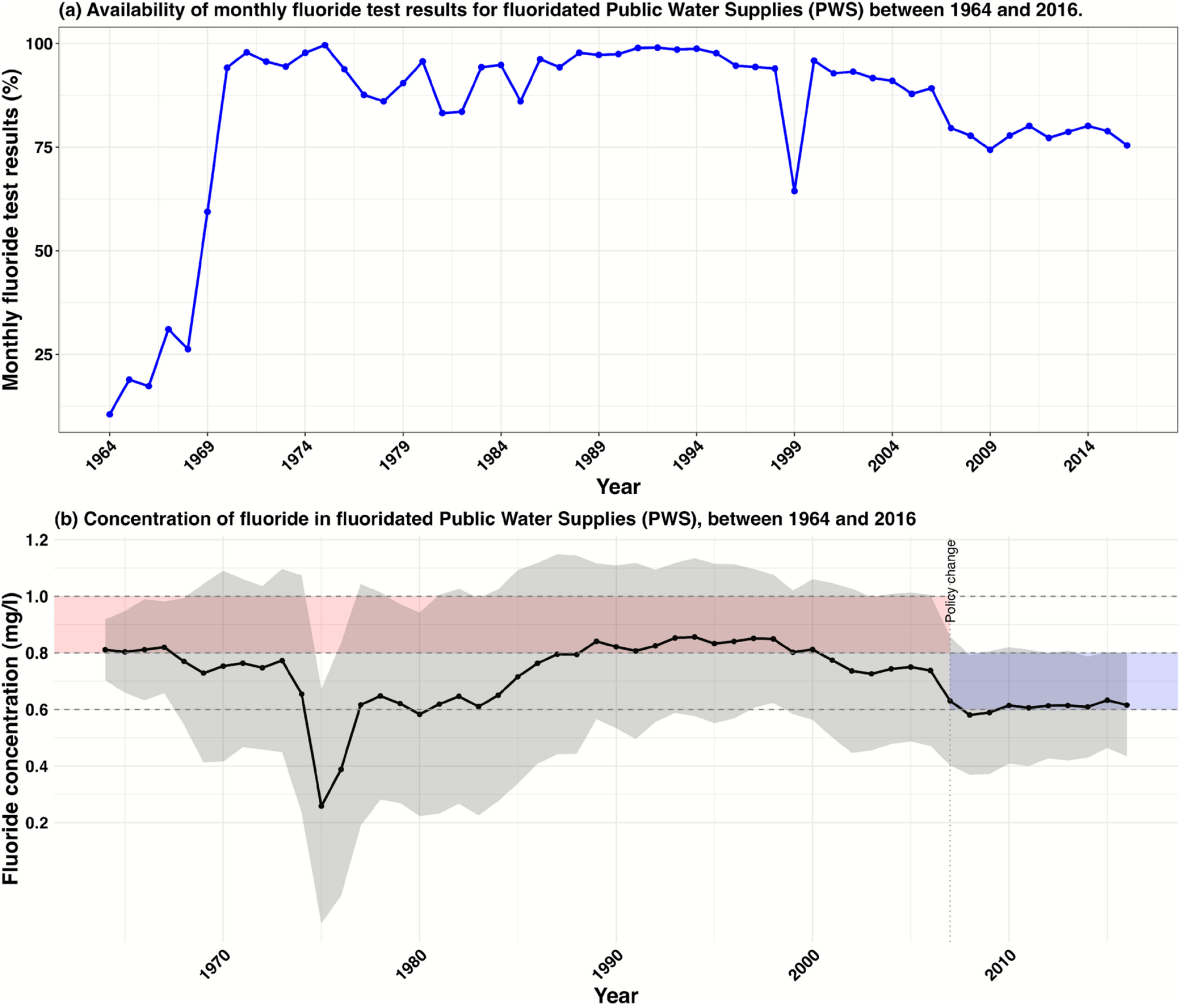
(a) Availability of monthly fluoride test results for fluoridated Public Water Supplies (PWS) between 1964 and 2016; (b) Concentration of fluoride in fluoridated Public Water Supplies (PWS), between 1964 and 2016. Annual mean fluoride (black line) and mean ± SD (shaded grey areas). Target ranges: 0.8–1.0 mg/L (1964–2006) and 0.6–0.8 mg/L (2007–2016).

Between 1964 and 2016, 129 831 fluoride test results were collected, of which 15 626 results were missing (12%). The variation of missing results ranged between 1.7% (County Cavan) and 30% (County Laois) (Table S2, Data S1). Data availability improved substantially over the study period. By 1970, 95% of expected monthly fluoride test results were available. However, availability fluctuated in subsequent decades: approximately 85% during the 1970s–1990s, declining to 65% around 1999, before stabilising at 75% by 2016 (Figure 1a). Missing data showed no significant seasonal variation (*p* > 0.05).

#### 3.2.2 PWS Compliance

**FIGURE 2:**
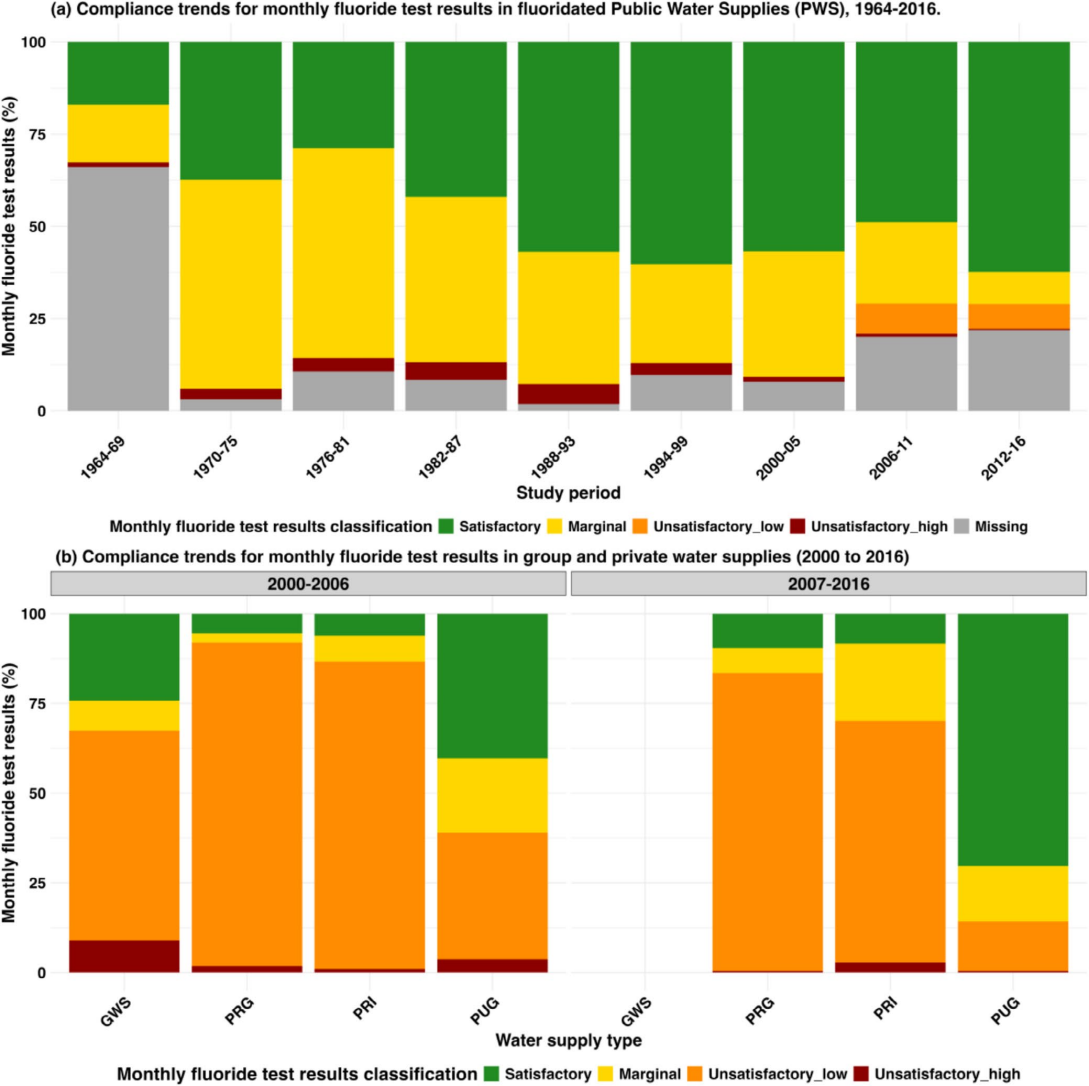
(a) Compliance trends for monthly fluoride test results in fluoridated Public Water Supplies (PWS) from 1964 to 2016 by compliance category. Target ranges: 0.8–1.0 mg/L (1964–2006); 0.6–0.8 mg/L (2007–2016); (b) Compliance trends for monthly fluoride test results in group and private water supplies from 2000 to 2016 by compliance category. Target range between 2000 and 2006: 0.8–1.0 mg/L; between 2007 and 2016: 0.6–0.8 mg/L. GWS (Group water supplies, could be private or public), PRG (Group water supplies with a private source), PRI (Private water supplies), PUG (Group water supplies with a public source). Statutory compliance standards apply only to fluoridated public water supplies and are not applicable to non‐fluoridated public, group or private supplies. For the purpose of comparative classification of fluoride concentrations, these thresholds were applied consistently across all water supply types, including public, group, and private water schemes.

**FIGURE 3:**
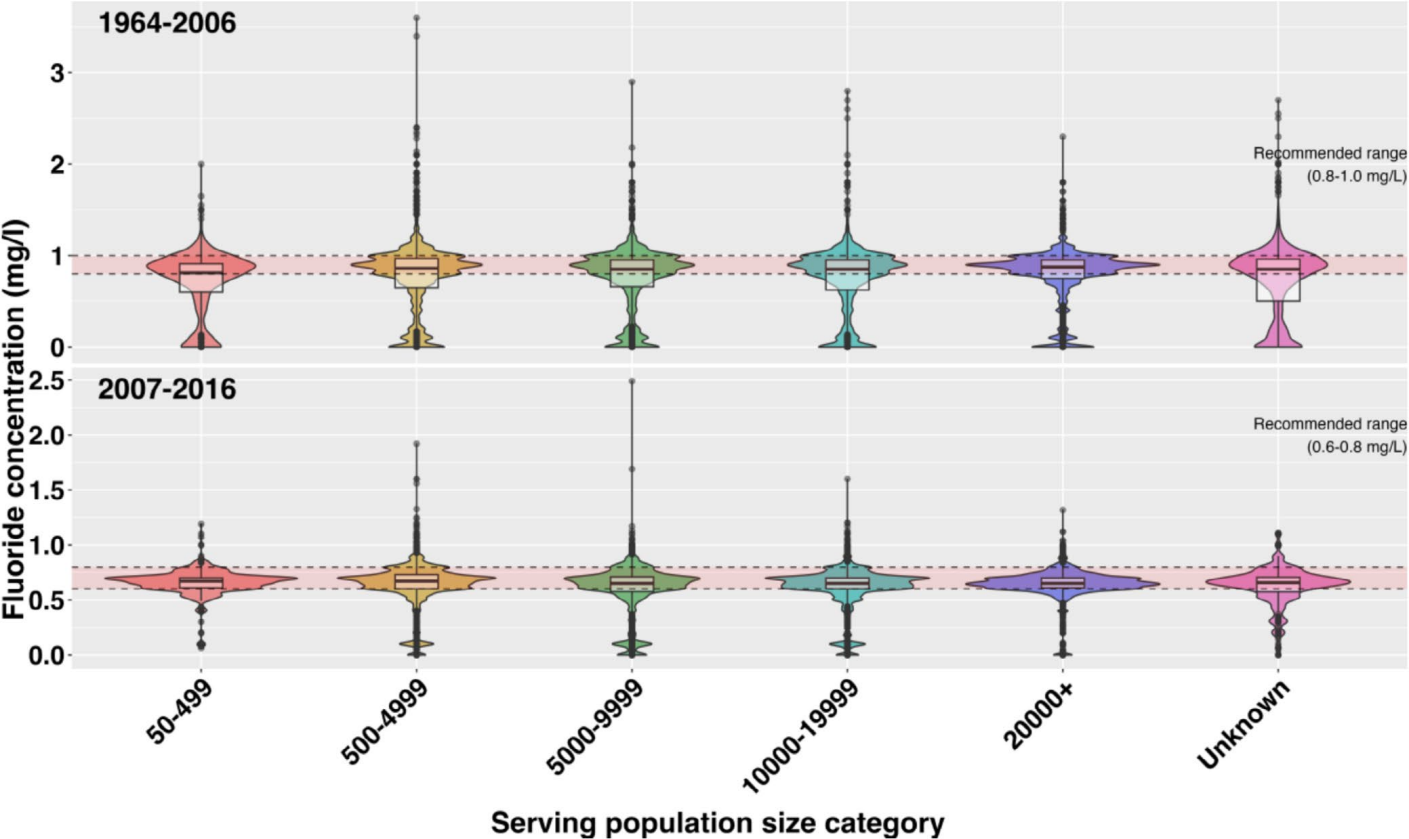
Fluoride concentrations in fluoridated Public Water Supplies (PWS) by population size and time period. Violin plots with box plots show fluoride distribution (mg/L) across population categories for 1964–2006 (upper) and 2007–2016 (lower). Recommended ranges: 0.8–1.0 mg/L (1964–2006) and 0.6–0.8 mg/L (2007–2016).

Between 1964 and 2006, *satisfactory* results increased progressively from 17% to 60% (1994–1999), before declining to 48% in 2006. *Marginal* results peaked at 57% (1976–81) then decreased, while *unsatisfactory high* results ranged between 1.2% and 5.5%. Following the 2007 target adjustment (0.6–0.8 mg/L), compliance improved across all categories. *Satisfactory* results increased from 49% to 62%, while *marginal* and *unsatisfactory* results decreased across all categories (Figure 2a and Table S3, Data S1). Mean fluoride concentration was 0.74 mg/L between 1964 and 2006, decreasing to 0.62 mg/L between 2007 and 2016, with reduced variability in the later period (SD 0.34 vs. 0.19) (Figure 1b and Table S4, Data S1). Across both time periods, there was relatively little variation in fluoride levels between different PWS categories from the smaller (50–499 people) to the larger (20 000+ people) PWS categories (Figure 3 and Tables S5 and S6, Data S1). In the 2007–2016 period, the larger PWS appear to cluster more consistently around the optimal fluoride concentration of 0.7 mg/L (Figure 3).

### 3.3 Fluoride Levels Across Group and Private Water Supplies

Between 2000 and 2006, group supplies with public sources (PUG) recorded 41% *satisfactory* results, while private supplies (PRI (Private water supplies) and PRG (Group water supplies with a private source)) recorded predominantly *marginal* results (> 90%). Between 2007 and 2016, PUG *satisfactory* results increased to 68%. Private supplies (PRG and PRI) showed high proportions of *unsatisfactory low* results (83% and 66%, respectively). *Unsatisfactory high* results were < 1% (PRG) and 3% (PRI) (Figure 2b and Table S7, Data S1).

## 4 Discussion

This is the first study to analyse fluoride trends across all Irish water supply types over five decades. Previous Irish studies examined only Public Water Supplies (PWS) over shorter periods [22, 24, 25]. International studies using similar monthly monitoring approaches have been conducted in the US [26, 28, 29], UK [30, 31, 32] and Brazil [33, 34, 35], though most used existing data while Brazilian studies involved primary data collection.

The observed patterns of data quality reflect the evolution of Irish fluoridation infrastructure and monitoring capacity. Early data gaps resulted from equipment failures, chemical supply shortages, distribution challenges to smaller supplies, and inconsistent sampling protocols [19, 25]. Notable disruptions included a 9‐month hydrofluorosilicic acid shortage in 1975 and supply interruptions during 1980–1982 [36].

Irish PWS compliance rates (70% within target range, 2012–2016) compare favourably with international standards: 76% in the USA [26] and 28%–78% in the UK [30]. However, Ireland shows notably lower exceedance rates (0.27% above 0.9 mg/L) compared to 7%–30% (USA) and 3%–24% (UK) above their respective thresholds, suggesting conservative dosing practices. This conservative approach may reflect Ireland's comprehensive legislative framework requiring mandatory fluoridation with regular monitoring. Methodological differences limit direct comparisons.

In contrast to the US study, which analysed monthly average fluoride readings (MAFRs) provided by state drinking water programmes, and the UK study, which utilised daily or weekly measurements recorded by the water companies, the present study used individual monthly samples for each supply collected by Environmental Health Officers and analysed by public laboratories. This sampling framework has constituted the statutory fluoridation monitoring method in Ireland since the 1960s [22]. Performance monitoring improved markedly in the final decade of the study period, coinciding with two policy changes. The Fluoridation of Water Supplies Regulations 2007 (S.I. No. 42 of 2007) reduced the fluoride target range from 0.8–1.0 to 0.6–0.8 mg/L, requiring more rigorous operational control [5]. Under the Water Services (No. 2) Act 2013 [37], Uisce Éireann (Irish Water) consolidated responsibility for public water supplies in 2014, transferring oversight from local authorities into a single national utility with standardised protocols and centralised management. While the present analysis extends only through 2016, limiting assessment of Uisce Éireann's full impact, these regulatory and structural changes likely contributed to enhanced monitoring practices.

Proposed characteristics that are important in maintaining the recommended levels of fluoride include an increase in the size of population served, source of public water supply, an increase in tenure of the chief operator of the water supply because of internal consistency, stable staff, and natural fluoride content nearer the legal range [28]. However, the applicability of these factors to the Irish context is uncertain. PWS serving populations of more than 5000 people demonstrated more consistent fluoride control, consistent with international experience [26, 30]. This reflects better staffing, automated monitoring technology, and more rigorous quality protocols in larger systems [30].

Group water supplies with public sources matched PWS performance, as both receive water from Uisce Éireann [11]. However, private water supplies and group supplies with private sources showed very low natural fluoride levels. Before introducing water fluoridation, fluoride levels in more than 660 public water supplies were analysed, and only 5 exceeded 0.3 mg/L [22]. More recently, raw water sampling by Irish Water and EPA audits have confirmed that the fluoride levels in naturally occurring water were generally below < 0.3 mg/L [11, 21]. These findings demonstrate that Irish waters have consistently low baseline fluoride levels regardless of source type.

A recent systematic review [38] examined national, regional, and county‐level dental health surveys of Irish children from 1950 to 2021 summarising dental caries trends over the last seven decades. The review reported substantial reductions in dental caries prevalence over time, with greater reductions in fluoridated areas than in non‐fluoridated ones, suggesting water fluoridation is an effective intervention for preventing dental caries in the Irish population. CWF started in Dublin in 1964, and between 1961 and 2014, the mean dmft/DMFT (decayed, missing and filled teeth) scores among 5 and 12‐year‐olds living in County Dublin decreased by approximately 88% and 90% respectively [38]. While most of the population receives optimally fluoridated water through PWS, approximately 15% rely on private supplies with suboptimal fluoride levels. Private water supplies are predominantly located in rural and remote areas where connection to public water connections is unavailable or economically unfeasible [14, 22]. These areas face multiple disadvantages that compound oral health risks. Rural populations have limited access to dental services due to geographic barriers and lower dentist‐to‐population ratios [39]. Rural communities also typically have lower income and education levels, both established risk factors for poor oral health [39]. The concentration of private water supplies in already disadvantaged areas suggests that improving fluoride access alone may be insufficient [40]. Importantly, dental caries levels have declined even in non‐fluoridated areas of Ireland, largely due to fluoridated toothpaste use and the “halo effect” from consuming foods and beverages processed with fluoridated water [41]. A comprehensive approach to oral health disparities should address multiple factors: alternative fluoride delivery methods, improved dental service access, oral health promotion, and broader social determinants of health in these underserved communities.

This is the first comprehensive analysis of community water fluoridation trends across all Irish water supply types over five decades (1964–2016). The categorical classification system (*satisfactory/marginal/unsatisfactory low/unsatisfactory high*) provides more meaningful insights than simple means, which can be misleading due to missing data and high variability [42]. Population‐based stratification revealed important patterns in fluoridation coverage and compliance that aggregate data would mask. Additionally, including private and group water supplies demonstrates disparities in fluoride access in populations not covered under the statutory fluoride legislation. However, there were some data and methodological limitations. Key data limitations include 12% missing data with substantial geographic variation (1.7%–30%), exclusion of small PWS (< 500 people) due to limited data availability, and absence of data from approximately 170 000 household wells which remain exempt from regulatory oversight. These constraints may affect trend interpretation and limit generalisability to the full population. Additionally, the unavailable geographic boundary data limited assessment of county‐level patterns. An important methodological limitation is that the environmental, political, and operational factors influencing fluoridation implementation and compliance were not investigated.

Future research should focus on longitudinal monitoring to identify compliance challenges, factors driving successful fluoridation maintenance, and operational barriers faced by water treatment facilities. Qualitative studies exploring public awareness and community perceptions of fluoridation would inform implementation strategies. An annually updated national fluoride database is needed, managed by a public health body or an academic institution, similar to that in Australia [43]. Longitudinal supply coverage maps should be accessible to researchers to monitor changes in fluoridation levels. Additionally, fluoridation monitoring data should be made publicly available, enabling communities to advocate for improved performance when local water fails to meet target fluoride levels. A community water fluoridation flagging system, similar to the UK model, would improve identification of fluoridated supply zones [31]. Comparative analyses with international programs could identify best practices for implementation and monitoring.

## 5 Conclusions

This analysis demonstrated progressive improvement in the CWF programme in Ireland, with compliance rates reaching 70% by 2016. There has been marked improvement in fluoride control in PWS over the decades, with the majority of PWS across all population categories consistently maintaining fluoride concentrations within recommended limits. The natural fluoride levels in group water supplies with a private source and small private supplies were suboptimal. In areas where optimal fluoridation is not achievable, alternative fluoride delivery methods should be considered.

## Author Contributions

Conceptualisation: V.S., M.C. and B.O.; Methodology: V.S., M.C., E.O., J.R. and B.O.; Data acquisition and statistical analysis: V.S., E.O., J.R., M.C., M.C., B.O. and O.C.; Interpretation of findings: V.S., M.C., M.C., B.O. and L.W.; Drafting and critically reviewing the article: V.S., M.C., M.O., M.C., L.W., J.M. and B.O.; Project administration: B.O. All authors have read and approved the final article.

## Funding

The Health Research Board in Ireland supported this study. Research grant number: APA‐2016‐1882.

## Ethics Statement

The authors have nothing to report.

## Consent

The authors have nothing to report.

## Conflicts of Interest

The authors declare no conflicts of interest.

## Supporting information


**Figure S1:** Distribution of Public Water Supplies by population serving size.
**Table S1:**. Types of drinking water supplies in Ireland.
**Table S2:** Missing fluoride testing results by county.
**Tables S3–S6:** Percentage distribution and descriptive summaries of monthly fluoride test results in fluoridated Public Water Supplies.
**Table S7:** Percentage distribution of monthly fluoride test results in group and private water supplies.

## Data Availability

All data generated and analysed during this study are not publicly available but can be made available on request from the corresponding author.
