# Supplementary material for: Longitudinal Analysis of Fluoride Levels in Irish Water Supplies: A 52‐Year Review
Source: Community Dent Oral Epidemiol. 2026 Jan 28;54(4):418–26. doi: 10.1111/cdoe.70055 (PMC13428059; doi:10.1111/cdoe.70055)
Supplement: Supplementary file 1 — Figure S1: Distribution of Public Water Supplies by population serving size. Table S1:. Types of drinking water supplies in Ireland. Table S2: Missing fluoride testing results by county. Tables S3–S6: Percentage distribution and descriptive summaries of monthly fluoride test results in fluoridated Public Water Supplies. Table S7: Percentage distribution of monthly fluoride test results in group and private water supplies. [file CDOE-54-418-s001.docx]

**Supplementary file S1**

Table S1 Types of drinking water supplies in Ireland, adapted from ^1^

| **Water supply type** | **Description** | **Supervising authority** | **Raw water source** | **Monitoring authority** | **Applicable standard for fluoride in drinking water** | **Number of supplies (% Irish population served in 2015)** |
| --- | --- | --- | --- | --- | --- | --- |
| Public water supplies | The abstraction, treatment and distribution of treated water managed by Uisce Éireann. These supplies are covered by the Drinking Water Regulations | EPA | Surface water, some groundwater | Uisce Éireann | 0.8 mg/L | 962 (83.3) |
| Public group schemes | The abstraction and treatment of water managed by Uisce Éireann. The distribution of treated water is managed by group water scheme, set up by the local community | Local authorities | Public mains (surface/ground) | Local authorities on behalf of their owners or trustees. | 1.5 mg/L | 498 (1.8) |
| Private group schemes | The abstraction, treatment and distribution of treated water is managed by group water scheme, set up by the local community | Local authorities | Wells, springs, local lakes/rivers | Local authorities on behalf of their owners or trustees. Some of them employ the HSE to test the water quality and some do it themselves. | 1.5 mg/L | 418 (4.2) |
| Small private supplies | Serving a commercial or public activity (e.g., hotels, pubs and national schools etc.). The abstraction, treatment and distribution of treated water managed by the manager or owner of the activity | Local authorities | Wells/ springs | Local authorities on behalf of their owners or trustees. | 1.5 mg/L | 1,760 (0.9) |
| Household wells (Exempted supplies) * | Mostly in rural areas serving private homes. The supply is managed by the household owner | Exempted | Wells | Owner of the supply or well. | Exempted | 170,000 (9.8) |

* *Exempted supply refers to a supply of water that (a) (i) is an individual supply of less than 10 cubic metres per day on average or serves fewer than 50 persons, and (ii) is not provided as part of a commercial or public activity, or (b) is used solely for purposes where the relevant supervisory authority is satisfied that the quality of the water does not have a direct or indirect influence on the health of the consumers concerned. ^2,3^*

Table S2 Percentage of missing fluoride testing results by county between 1964 and 2016.

| **County** | **Percentage of missing fluoride results** |
| --- | --- |
| Cavan | 1.67 |
| Monaghan | 3.36 |
| Longford | 3.37 |
| Cork | 4.06 |
| Westmeath | 4.25 |
| Sligo | 4.76 |
| Roscommon | 5.35 |
| Kerry | 5.73 |
| Limerick | 5.88 |
| Galway | 6.02 |
| Mayo | 6.54 |
| Clare | 7.05 |
| Donegal | 7.07 |
| Tipperary- North | 11.44 |
| Kildare | 11.53 |
| Wicklow | 11.71 |
| Dublin | 13.37 |
| Louth | 18.31 |
| Carlow | 21.89 |
| Tipperary- South | 22.21 |
| Kilkenny | 22.67 |
| Offaly | 23.00 |
| Meath | 23.96 |
| Wexford | 24.27 |
| Waterford | 24.46 |
| Laois | 30.02 |

Table S3 Percentage distribution of monthly fluoride test results in Irish fluoridated public water supplies from 1964 to 2016, by compliance category. Recommended fluoride range: 0.8 to 1.0 mg/l (from 1964 to 2007) and 0.6 to 0.8 mg/L (from 2007 to 2016).

| **Period**  *Recommended F level: 0.80 – 1.0 mg/L* | **Valid (Non-missing) %** | **Satisfactory**  (0.80 to 1.00) mg/L  **%** | **Marginal**  (0.70<0.80) & (>1.00 ≤1.10) mg/L  **%** | **Unsatisfactory low**  (<0.70) mg/L  % | **Unsatisfactory high**  (≥1.11) mg/L  **%** |
| --- | --- | --- | --- | --- | --- |
| 1964-69 | 66.11 | 17.01 | 15.70 | 0 | 1.18 |
| 1970-75 | 3.20 | 37.37 | 56.66 | 0 | 2.77 |
| 1976-81 | 10.63 | 28.85 | 56.83 | 0 | 3.68 |
| 1982-87 | 8.37 | 41.98 | 44.88 | 0 | 4.76 |
| 1988-93 | 1.83 | 56.86 | 35.83 | 0 | 5.48 |
| 1994-99 | 9.75 | 60.29 | 26.79 | 0 | 3.18 |
| 2000-05 | 7.91 | 56.76 | 33.98 | 0 | 1.35 |
| 2006 | 10.80 | 47.87 | 40.01 | 0 | 1.32 |
| **Period**  *Recommended F level: 0.60 -0.80 mg/L* | **Missing**  **%** | **Satisfactory**  (0.60 to 0.80) mg/L  % | **Marginal**  (0.50<0.60) & (>8.00 ≤0.90) mg/L  % | **Unsatisfactory low**  (<0.50) mg/L  % | **Unsatisfactory high**  (≥0.90) mg/L  % |
| 2007-11 | 22.09 | 48.68 | 15.90 | 11.34 | 1.99 |
| 2012-16 | 21.94 | 62.38 | 8.67 | 6.74 | 0.27 |

Table S4 Descriptive summaries of the monthly fluoride (mg/L) testing results in fluoridated public water supplies from the 1960s to 2016.

| **Study period** | **Valid reasults** | **Mean** | **SD** | **Median** | **Q1**  **Q_1_** | **Q3**  **Q_3_** | **Min** | **Max** |
| --- | --- | --- | --- | --- | --- | --- | --- | --- |
| 1960s - 2016 | 114,200 | 0.71 | 0.32 | 0.80 | 0.61 | 0.91 | 0 | 3.6 |
| 1960s - 2006 | 89,324 | 0.74 | 0.34 | 0.86 | 0.67 | 0.95 | 0 | 3.6 |
| 2007-2016 | 24,876 | 0.62 | 0.19 | 0.66 | 0.6 | 0.71 | 0 | 2.49 |

Table S5 Descriptive summaries of the monthly fluoride (mg/L) testing results in fluoridated public water supplies from the 1960s to 2016, by serving population size category and period.

| **Period** | **Population serving size** | **Valid results** | **Mean** | **SD** | **Median** | **Q_1_** | **Q_3_** | **Min** | **Max** |
| --- | --- | --- | --- | --- | --- | --- | --- | --- | --- |
| 1964-69 | 50-499 | 60 | 0.71 | 0.25 | 0.80 | 0.60 | 0.87 | 0.05 | 1.15 |
| 1964-69 | 500-4999 | 154 | 0.56 | 0.47 | 0.76 | 0.00 | 0.95 | 0.00 | 1.43 |
| 1964-69 | 5000-9999 | 89 | 0.70 | 0.37 | 0.82 | 0.50 | 0.99 | 0.00 | 1.20 |
| 1964-69 | 10000-19999 | 234 | 0.84 | 0.25 | 0.90 | 0.75 | 1.00 | 0.00 | 1.35 |
| 1964-69 | 20000+ | 217 | 0.74 | 0.32 | 0.85 | 0.70 | 0.95 | 0.00 | 1.10 |
| 1964-69 | Unknown | 77 | 0.77 | 0.34 | 0.90 | 0.70 | 0.95 | 0.00 | 1.40 |
| 1970-75 | 50-499 | 209 | 0.64 | 0.35 | 0.75 | 0.43 | 0.90 | 0.00 | 1.50 |
| 1970-75 | 500-4999 | 2,133 | 0.60 | 0.43 | 0.75 | 0.05 | 0.95 | 0.00 | 1.70 |
| 1970-75 | 5000-9999 | 1,225 | 0.58 | 0.43 | 0.75 | 0.00 | 0.95 | 0.00 | 1.80 |
| 1970-75 | 10000-19999 | 1,345 | 0.62 | 0.42 | 0.80 | 0.07 | 0.95 | 0.00 | 1.50 |
| 1970-75 | 20000+ | 1,988 | 0.68 | 0.38 | 0.83 | 0.45 | 0.98 | 0.00 | 1.50 |
| 1970-75 | Unknown | 1,150 | 0.57 | 0.44 | 0.75 | 0.00 | 0.98 | 0.00 | 1.50 |
| 1976-81 | 50-499 | 237 | 0.59 | 0.36 | 0.70 | 0.30 | 0.81 | 0.00 | 1.45 |
| 1976-81 | 500-4999 | 3,401 | 0.54 | 0.43 | 0.63 | 0.00 | 0.90 | 0.00 | 2.00 |
| 1976-81 | 5000-9999 | 1,461 | 0.58 | 0.42 | 0.70 | 0.10 | 0.93 | 0.00 | 1.80 |
| 1976-81 | 10000-19999 | 1,709 | 0.53 | 0.43 | 0.65 | 0.00 | 0.90 | 0.00 | 2.70 |
| 1976-81 | 20000+ | 2,403 | 0.64 | 0.39 | 0.80 | 0.26 | 0.95 | 0.00 | 1.50 |
| 1976-81 | Unknown | 1,730 | 0.47 | 0.46 | 0.35 | 0.00 | 0.93 | 0.00 | 2.30 |
| 1982-87 | 50-499 | 275 | 0.63 | 0.38 | 0.80 | 0.21 | 0.90 | 0.00 | 2.00 |
| 1982-87 | 500-4999 | 4,234 | 0.67 | 0.41 | 0.80 | 0.20 | 0.97 | 0.00 | 3.60 |
| 1982-87 | 5000-9999 | 2,064 | 0.73 | 0.36 | 0.85 | 0.56 | 1.00 | 0.00 | 1.80 |
| 1982-87 | 10000-19999 | 2,048 | 0.70 | 0.38 | 0.80 | 0.46 | 1.00 | 0.00 | 2.80 |
| 1982-87 | 20000+ | 2,580 | 0.79 | 0.32 | 0.90 | 0.72 | 1.00 | 0.00 | 2.30 |
| 1982-87 | Unknown | 2,072 | 0.70 | 0.40 | 0.80 | 0.27 | 1.00 | 0.00 | 2.70 |
| 1988-93 | 50-499 | 330 | 0.74 | 0.35 | 0.85 | 0.70 | 0.95 | 0.00 | 1.65 |
| 1988-93 | 500-4999 | 5,638 | 0.81 | 0.31 | 0.90 | 0.74 | 1.00 | 0.00 | 3.40 |
| 1988-93 | 5000-9999 | 3,129 | 0.84 | 0.29 | 0.90 | 0.80 | 1.00 | 0.00 | 2.90 |
| 1988-93 | 10000-19999 | 2,312 | 0.82 | 0.29 | 0.90 | 0.79 | 1.00 | 0.00 | 2.00 |
| 1988-93 | 20000+ | 2,750 | 0.84 | 0.25 | 0.90 | 0.80 | 0.96 | 0.00 | 1.80 |
| 1988-93 | Unknown | 2,496 | 0.80 | 0.34 | 0.90 | 0.72 | 1.00 | 0.00 | 2.55 |
| 1994-99 | 50-499 | 260 | 0.92 | 0.16 | 0.93 | 0.90 | 1.00 | 0.10 | 1.30 |
| 1994-99 | 500-4999 | 6,645 | 0.84 | 0.28 | 0.90 | 0.80 | 1.00 | 0.00 | 2.35 |
| 1994-99 | 5000-9999 | 3,293 | 0.81 | 0.29 | 0.89 | 0.78 | 0.96 | 0.00 | 2.18 |
| 1994-99 | 10000-19999 | 2,299 | 0.83 | 0.26 | 0.90 | 0.80 | 0.99 | 0.00 | 2.50 |
| 1994-99 | 20000+ | 2,728 | 0.85 | 0.21 | 0.90 | 0.83 | 0.95 | 0.00 | 1.45 |
| 1994-99 | Unknown | 2,426 | 0.82 | 0.27 | 0.89 | 0.79 | 0.96 | 0.00 | 2.50 |
| 2000-05 | 50-499 | 345 | 0.75 | 0.29 | 0.85 | 0.71 | 0.91 | 0.00 | 1.34 |
| 2000-05 | 500-4999 | 7,709 | 0.78 | 0.27 | 0.86 | 0.75 | 0.93 | 0.00 | 2.33 |
| 2000-05 | 5000-9999 | 3,541 | 0.71 | 0.31 | 0.81 | 0.66 | 0.90 | 0.00 | 1.80 |
| 2000-05 | 10000-19999 | 2,425 | 0.75 | 0.27 | 0.83 | 0.72 | 0.90 | 0.00 | 1.22 |
| 2000-05 | 20000+ | 3,073 | 0.80 | 0.20 | 0.85 | 0.78 | 0.90 | 0.00 | 1.52 |
| 2000-05 | Unknown | 1,791 | 0.76 | 0.26 | 0.82 | 0.71 | 0.90 | 0.00 | 1.81 |
| 2006-11 | 50-499 | 298 | 0.62 | 0.20 | 0.65 | 0.55 | 0.71 | 0.00 | 1.10 |
| 2006-11 | 500-4999 | 6,328 | 0.65 | 0.23 | 0.69 | 0.60 | 0.80 | 0.00 | 1.84 |
| 2006-11 | 5000-9999 | 3,319 | 0.60 | 0.25 | 0.66 | 0.54 | 0.75 | 0.00 | 1.28 |
| 2006-11 | 10000-19999 | 2,226 | 0.64 | 0.22 | 0.67 | 0.59 | 0.76 | 0.00 | 1.60 |
| 2006-11 | 20000+ | 2,873 | 0.67 | 0.17 | 0.67 | 0.61 | 0.76 | 0.00 | 1.32 |
| 2006-11 | Unknown | 612 | 0.65 | 0.25 | 0.68 | 0.55 | 0.80 | 0.00 | 1.20 |
| 2012-16 | 50-499 | 248 | 0.68 | 0.08 | 0.68 | 0.64 | 0.71 | 0.10 | 1.19 |
| 2012-16 | 500-4999 | 5,022 | 0.64 | 0.17 | 0.67 | 0.61 | 0.72 | 0.00 | 1.92 |
| 2012-16 | 5000-9999 | 2,792 | 0.60 | 0.21 | 0.65 | 0.60 | 0.70 | 0.00 | 2.49 |
| 2012-16 | 10000-19999 | 1,760 | 0.60 | 0.19 | 0.65 | 0.60 | 0.70 | 0.00 | 0.90 |
| 2012-16 | 20000+ | 2,302 | 0.65 | 0.08 | 0.65 | 0.61 | 0.70 | 0.00 | 1.00 |
| 2012-16 | Unknown | 135 | 0.65 | 0.07 | 0.66 | 0.62 | 0.70 | 0.35 | 0.81 |

Table S6 Descriptive summaries of the monthly fluoride testing results in Irish fluoridated public water supplies from the 1960s to 2016 by the population served size.

| **Population category** | **Valid reasults** | **Mean** | **SD** | **Median** | **Q1** | **Q3** | **Min** | **Max** |
| --- | --- | --- | --- | --- | --- | --- | --- | --- |
| 50-499 | 2,262 | 0.70 | 0.30 | 0.77 | 0.60 | 0.90 | 0 | 2.0 |
| 500-4999 | 41,264 | 0.71 | 0.32 | 0.80 | 0.61 | 0.91 | 0 | 3.6 |
| 5000-9999 | 20,913 | 0.70 | 0.32 | 0.78 | 0.60 | 0.90 | 0 | 2.9 |
| 10000-19999 | 16,358 | 0.70 | 0.32 | 0.80 | 0.60 | 0.90 | 0 | 2.8 |
| 20000+ | 20,914 | 0.74 | 0.27 | 0.81 | 0.65 | 0.91 | 0 | 2.3 |
| Unknown | 12,489 | 0.70 | 0.36 | 0.83 | 0.52 | 0.95 | 0 | 2.7 |

Table S7 Percentage distribution of monthly fluoride test results in group and private water supplies from 1964 to 2016, by compliance category. Recommended range: 0.8 to 1.0 mg/L (from 2000 to 2007) and 0.6 to 0.8 mg/L (from 2007 to 2016).

| **Supply type** | **Total number of Fluoride**  **Samples** | **Satisfactory**  (0.80 to 1.00) mg/L | **Marginal**  (0.70<0.80) & (>1.00 ≤1.10) mg/L | **Unsatisfactory low**  (<0.70) mg/L | **Unsatisfactory high**  (≥1.11) mg/L |
| --- | --- | --- | --- | --- | --- |
| *2000- 2006 Recommended F level: 0.8 – 1.0 mg/L)* | | | | | |
| GWS (n = 275) | 338 | 24.26 | 66.86 | 0 | 8.87 |
| PRG (n = 453) | 889 | 5.51 | 92.57 | 0 | 1.91 |
| PRI (n = 59) | 85 | 4.70 | 94.11 | 0 | 1.18 |
| PUG (n = 392) | 681 | 41.40 | 54.18 | 0 | 4.4 |
| *2007-2016 Recommended F level: 0.6 -08 mg/L* | | | | | |
| **Supply type** | **Total number of Fluoride**  **Samples** | **Satisfactory**  (0.60 to 0.80) mg/L | **Marginal**  (0.50<0.60) & (>8.00 ≤0.90) mg/L | **Unsatisfactory low**  (<0.50) mg/L | **Unsatisfactory high**  (≥0.90) mg/L |
| PRG (n = 429) | 2169 | 9.49 | 6.91 | 83.17 | 0.4 |
| PRI (n = 120) | 338 | 8.87 | 22.19 | 66.27 | 2.66 |
| PUG (n = 433) | 2514 | 68.13 | 16.43 | 13.8 | 1.63 |

Note: Statutory compliance standards apply only to fluoridated public water supplies and are not applicable to non-fluoridated public, group or private supplies. For the purpose of comparative classification of fluoride concentrations, these thresholds were applied consistently across all water supply types, including public, group, and private water schemes.


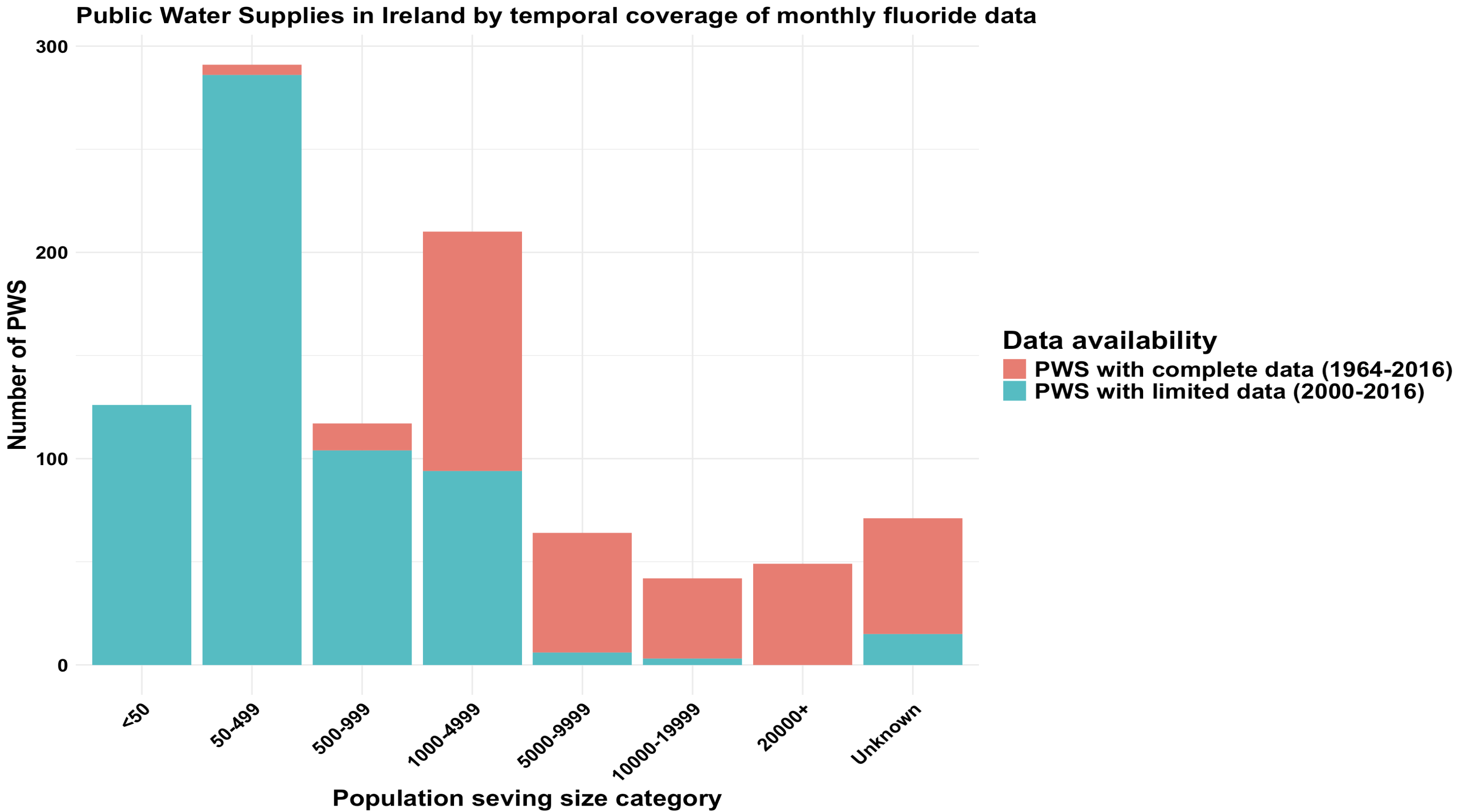


Figure S1 Number of public water supplies in Ireland by population serving size and temporal coverage of monthly fluoride testing results data.

**Reference:**

1. Roche M, Page D, Gillard P, Devaney D, Dunne N, Barrington R. *Drinking Water Report for Public Supplies 2015*. 2016. Accessed 11/02/2025. <https://www.epa.ie/publications/compliance--enforcement/drinking-water/2015-DW-Report-Public-Supplies_web.pdf>

2. European Communities (Drinking Water) Regulations, 2000., Government of Ireland. (2000). <https://www.irishstatutebook.ie/eli/2000/si/439/made/en/print#article1>

3. European Union (Drinking Water) Regulations 2023, Government of Ireland (Government of Ireland 2023). Accessed 09/03/2025. <https://www.irishstatutebook.ie/eli/2023/si/99/made/en/print>
